# Damage control resuscitation: lessons learned

**DOI:** 10.1007/s00068-015-0628-3

**Published:** 2016-02-04

**Authors:** M. Giannoudi, P. Harwood

**Affiliations:** Leeds General Infirmary, Clarendon Wing, Level A, Great George Street, Leeds, West Yorkshire LS1 3EX UK

**Keywords:** Polytrauma, Damage control surgery, Severe haemorrhage, Resuscitation

## Abstract

**Background:**

Damage control resuscitation describes an approach to the early care of very seriously injured patients. The aim is to keep the patient alive whilst avoiding interventions and situations that risk worsening their situation by driving the lethal triad of hypothermia, coagulopathy and acidosis or excessively stimulating the immune-inflammatory system. It is critical that the concepts and practicalities of this approach are understood by all those involved in the early management of trauma patients. This review aims to summarise this and discusses current knowledge on the subject.

**Interventions:**

Damage control resuscitation forms part of an overall approach to patient care rather than a specific intervention and has evolved from damage control surgery. It is characterised by early blood product administration, haemorrhage arrest and restoration of blood volume aiming to rapidly restore physiologic stability. The infusion of large volumes of crystalloid is no longer appropriate, instead the aim is to replace lost blood and avoid dilution and coagulopathy. In specific situations, permissive hypotension may also be of benefit, particularly in patients with severe haemorrhage from an arterial source. As rapid arrest of haemorrhage is so important, team-based protocols that deliver patients rapidly but safely, via CT scan where appropriate, to operating theatres or interventional radiology suites form a critical part of this process.

**Conclusions:**

Given that interventions are so time dependent in the severely injured, it is likely that by further improving trauma systems and protocols, improvements in outcome can still be made. Further research work in this area will allow us to target these approaches more accurately to those patients who can benefit most.

## Introduction

The term “damage control” originates in naval conflict, being used to describe an approach to managing damaged warships that aims to maintain seaworthiness and operational status rather than repair all damage [1, 2]. The concept has been transferred into medical practice and used to describe a management approach in severely and multiply injured patients. This is relevant to the widely popularised two hit model of the systemic inflammatory response to trauma which hypothesises that serious injury may lead to excessive activation of the immune-inflammatory system (the first hit). Whilst in this state, which is time dependent, further insult (the second hit), including surgical intervention, can tip the balance pushing patients into a state of uncontrolled physiological disequilibrium (termed the systemic inflammatory response syndrome). The initial pathophysiologic response to severe injury is characterised by the classically described lethal triad of hypothermia, coagulopathy and acidosis. These features together form a progressive cycle with resultant exacerbation of haemorrhage driving the patients physiology further from normal. This situation is often rapidly fatal unless corrected. Even when the patient survives the initial phase, the result is frequently multiple organ dysfunction and damage including acute lung injury, renal and hepatic dysfunction, circulatory and immune failure. Such a situation is a potent cause of late morbidity and mortality in multiply injured patients.

Damage control surgery aims to reduce this effect by minimising the early surgical burden and therefore the second hit. The patient is then taken to critical care facilities for ongoing resuscitation and physiologic restoration. Once the initial phase has passed, staged anatomic reconstruction is undertaken to restore long term function. The intention is to avoid excessive surgical stress from lengthy or specifically stimulatory reconstructive interventions in patients who are physiologically not equipped to withstand them. Instead the focus is changed to undertake only interventions required to save life and limb and then stabilise the patients’ physiology, delaying definitive reconstruction until recovery has begun.

Damage control surgery was first developed in the management of abdominal injuries, particularly after gun shot wounds. This was defined as the ‘initial control of haemorrhage and contamination followed by intraperitoneal packing and rapid closure’ to allow for ‘resuscitation to normal physiology in the intensive care unit and a subsequent definitive re-exploration’ [3]. Subsequently this rationale was also applied to skeletal trauma (damage control orthopaedics, DCO). In this context the focus is on temporary skeletal stabilization, usually by external fixation, and management of soft tissue injury with definitive reconstruction once the patients physiology allows [1, 4–6].

It is recognised that stresses other than surgery can also contribute to this process of disordered immune activation, including episodes of hypotension, hypoxia and relative tissue ischaemia as well as early complications such as infection [7, 8]. Thus, it can be seen that this treatment rationale should be applied holistically to the patient if its benefits are to be realised. Recently therefore, greater attention has been given to the resuscitative phase of management in multiply injured patients. Clinical understanding has expanded greatly from modern military conflict, these advances are now being transferred to civilian practice. The development of regional and national trauma systems with resultant concentration of severely injured patients has further increased experience. Ongoing basic science and clinical research has lead to an increasing understanding of these processes at a molecular level. Damage control resuscitation represents evidence based evolution of different resuscitation protocols that have been developed over many years [1, 2].

This paper aims to review current practice in the resuscitation of multiply and severely injured patients and document lessons leaned thus far when this approach is used.

## Pathophysiology

### Hypothermia

Hypothermia is common among polytrauma patients, especially those with haemorrhagic shock. The causes are multiple but include physical exposure to the environment both at the scene and in hospital, intoxication, circulatory changes and administration of cold fluids [9]. The situation may be exacerbated by loss of thermoregulatory control due to uncoupling of normal metabolic pathways, resulting in the loss of homeothermic ability as physiologic derangement ensues.

Severe hypothermia is associated with a high mortality. Prognosis is directly related to the degree of hypothermia, with 100 % mortality having been observed in patients who present with core body temperatures under 32.8° [2]. Hypothermia causes and exacerbates bleeding abnormalities through multiple mechanisms. Moderate hypothermia (32–34 °C), directly reduces coagulation factor activity by approximately 10 % for each degree fall in temperature, whilst also inhibiting platelet aggregation [9, 10]. Decreased thromboxane activity potentiates bleeding, which is aggravated by dysregulation of coagulation factors and enzymes [11]. Steps should therefore be taken at every stage to prevent hypothermia and consideration given to rewarming those in which this has already occurred, this is discussed later in the review [2].

### Coagulopathy

Acute traumatic coagulopathy is a process recognised to occur early following the tissue trauma and shock resulting from severe injury. In polytrauma patients it has been associated with increased bleeding morbidity, higher transfusion requirements, increased risk of organ dysfunction, longer critical care stays and increased overall mortality [12–14]. The severity of post traumatic coagulopathy has been shown to directly correlate with overall injury severity [15]. Once established this state is extremely difficult to correct and is a potent cause of early death in such patients.

Historically, it was thought that coagulopathy in the polytrauma patient was due mainly to fluid administration and hypothermia, management was somewhat neglected [16, 17]. Administration of clotting products was only initiated once coagulopathy had ensued or large volumes of blood had been given, usually in excess of 10 units of red blood cells within 24 h. Large volumes of crystalloid solution were generally administered alongside packed red blood cells. It was recognised that this approach exacerbated coagulopathy and frequently lead to unsalvagable situations by further driving the lethal triad to exhaustion [15].

Coagulopathy of trauma arises from decreased circulating concentration, and dysfunction of, various components of the clotting mechanism. The incidence of coagulopathy appears to correlate directly with the volume of clear fluid administered supporting the view that dilution is contributory [18]. The ability to rapidly analyse different aspects of the clotting cascade has increased understanding of individual component dysfunction in this process. Hypovolaemic shock, reduced protein C levels and protein C activation have been observed in acute trauma [12]. This appears to result from the combined influence of acidosis, hemodilution and hypothermia [12]. Despite a paucity of research in this area it does appear that the coagulaopathy seen in acute trauma is driven by the activation of protein C and is associated with the depletion of many clotting factors including: I, II, V, VII, VIII, IX and X [14].

Two types of coagulopathy have been described; systemically acquired coagulopathy (SAC) and endogenous acute coagulaopathy (EAC). SAC is itself associated with the lethal triad of acidosis, hemodilution and hypothermia. EAC has been described as a precursor to SAC in the polytrauma patient with a coagulopathy related to the activation of the protein C pathway. In the presence of EAC mortality rates four times higher than in those with normal coagulation have been observed, along with increased transfusion requirements and rates of multiple organ dysfunction. The processes underlying these coagulopathic states are not fully understood and hence there are currently no firm definitions describing them [12].

### Acidosis

Inadequate or inappropriate circulation in trauma patients results from blood loss, tissue damage and vascular injury. This results in the generation of toxic metabolites, anaerobic metabolism and lactic acidosis. Homeostatic mechanisms carefully maintain a narrow pH range and as the patient deteriorates may become impaired themselves, exacerbating such problems. The degree of acidosis and lactate levels on admission have been shown to predict mortality in the trauma patient [11].

Alterations in pH will detrimentally affect enzymatic function throughout the body, resulting in multiple tissue and therefore organ dysfunction. Perhaps most importantly in the bleeding patient, the severity of acidosis has been shown to correlate with the dysfunction of coagulation factors [11]. A fall in pH from 7.4 to 7.0 reduces factor VIIa’s activity by 90 %, whilst the activity of factors Xa/Va have been shown to decrease by 70 % [2, 11]. Thus it can be seen that coagulopathic bleeding will worsen the state of shock, decreasing tissue perfusion and exacerbating acidosis and therefore coagulopathy. To address this, it is important to optimise oxygen delivery through blood transfusions and by augmenting cardiac output pharmacologically, whilst attempts are made to arrest haemorrhage as soon as possible [19]. This is especially evident at pH levels of 7.2 or less which have been associated with decreased cardiac contractility and cardiac output, vasodilation, hypotension, and bradycardias [2].

## Damage control resuscitation

Damage control resuscitation (DCR) forms part of an overall approach to patient care rather than a specific intervention and has evolved from damage control surgery. It is characterised by early blood product administration and haemorrhage control with restoration of blood volume and physiologic stability [1]. This approach should be initiated at first contact with the patient in the prehospital environment and continue through their initial reception and treatment until haemorrhage is arrested and physiology corrected. Recognition of patients at high risk is therefore critical. It has repeatedly been shown that early intervention to prevent physiologic derangement is far more effective than trying to correct self amplifying processes once they are established. Such an approach is most effective when delivered as part of entire package of care along with rapid surgery aimed at life and/or limb salvage whilst avoiding time consuming and potentially traumatic reconstruction. As rapid arrest of haemorrhage is so important, team based protocols that deliver patients rapidly but safely, via CT scan where appropriate, to operating theatres or interventional radiology suites form a critical part of this process. The main drive behind all of this is to try and avoid precipitating further pathophysiologic responses or amplifying those already underway [1, 20]. Guidelines for the use of DCR have now been implemented in many units and form the basis of early patient management protocols in major trauma centres [15].

### Indications for damage control

As DCR aims to pre-emptively address occult physiologic derangement to prevent catastrophic deterioration, early recognition of patients at risk is tantamount [2]. In the context of acute major trauma, all patients should initially be managed by a DCR approach. Such situations remain highly individual and dynamic, it is not sensible to construct strict criteria based protocols. It is quite reasonable to switch from one rationale to the other, responding to deterioration or improvement, though it is important to note the overall trend over time and adopt a damage control rationale where doubt exists [15]. Indeed it has been suggested that more widespread use of DCR might mean that damage control surgery may be required less often, as the patient is prevented from entering a pathophysiologic state in the first place.

A DCR approach should therefore be considered in all patients whose injuries place them at risk of significant haemorrhage or physiologic derangement. This would include those with or suspected of having major abdominal or thoracic visceral injury, significant pelvic trauma, significant amputation, multiple long bone fractures and head injuries. This should be escalated where patient physiology and biochemistry indicates that major blood loss is occurring or a pathophysiologic response is underway. The tables below suggest potential indicators of these events.

#### Damage control surgery

**Fig. 1:**
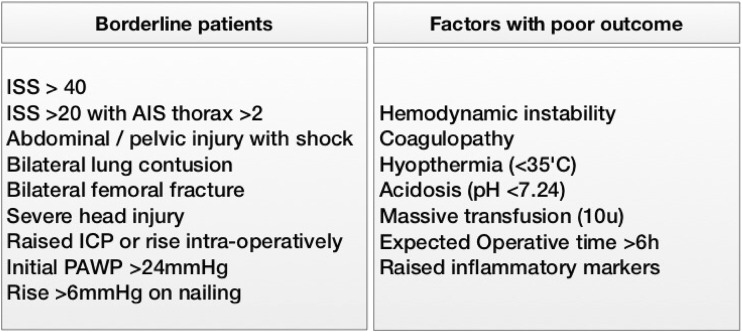
Defining borderline patients and factors associated with poor outcome in trauma patients—*ISS* injury severity score, *AIS* abbreviated injury scale, *ICP* intracranial pressure, *PAWP* pulmonary artery wedge pressure

**Fig. 2:**
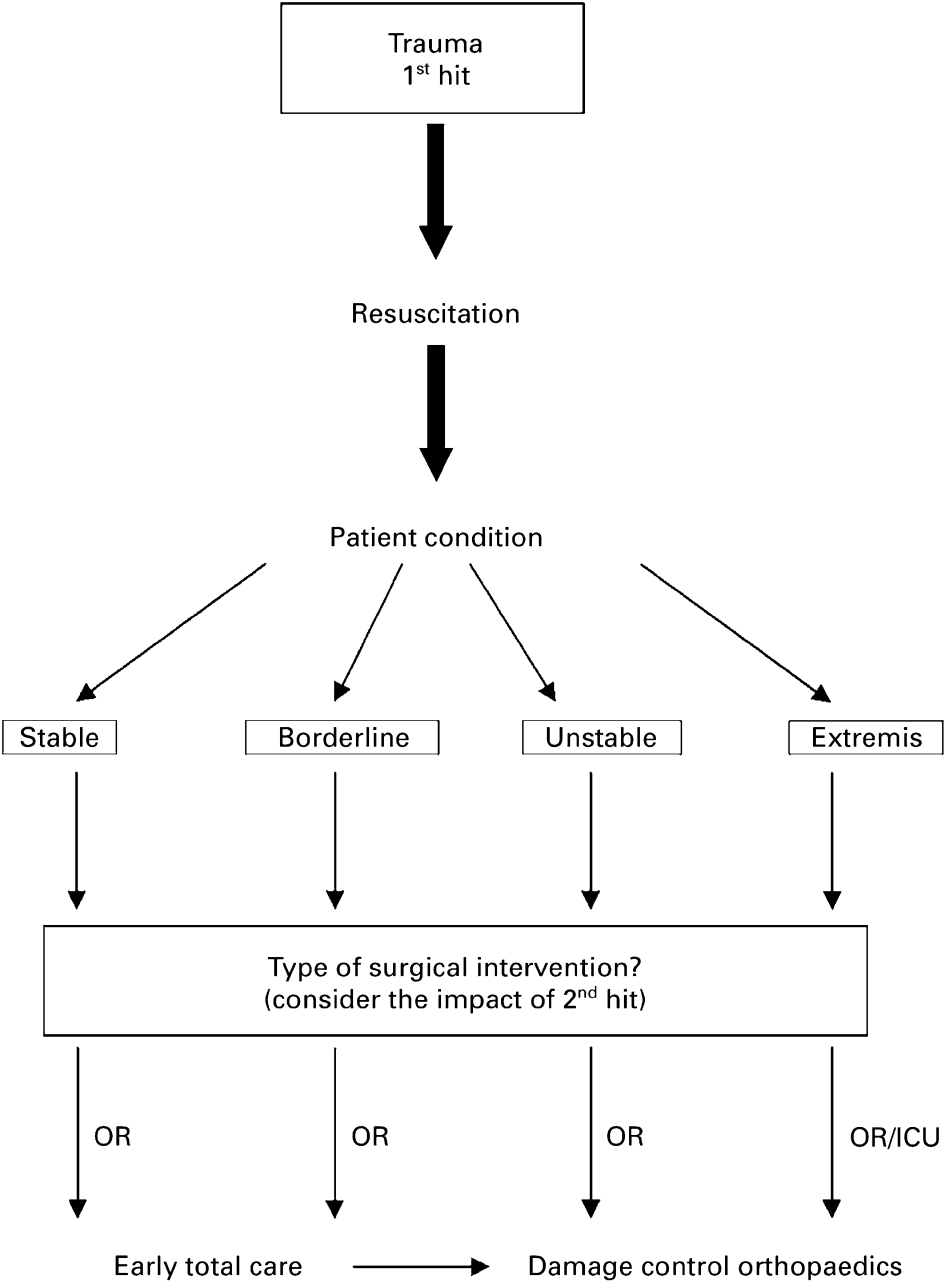
Diagram illustrating the concept of prioritisation and decision making in severely injured patients—*OR* operating room, *ICU* intensive care unit—reproduced with kind permission of author [21]

The Hanover group have developed of a set of sensible guidelines to aid decision making in these complex cases (Figs. 1, 2) [6]. This divides patients into four groups, those in extremis (peri-arrest with end stage signs of shock), haemodynamically unstable patients, borderline patients and stable patients. It is suggested that those in extremis and unstable patients are managed throughout utilising a damage control approach. Stable patients can be managed using standard protocols, the more difficult group are the borderline patients. These exhibit injury patterns or physiologic responses that are associated with poor outcome but have not been haemodynamically unstable or have responded to intervention and become stable. It is suggested that a flexible approach is adopted, with such patients being initially managed using standard protocols and having definitive reconstruction of injuries requiring surgery. Should such injury patterns be seen in conjunction with physiologic derangement consideration should be given to adopting a surgical damage control approach from the outset. Similarly, should there be any indication of physiologic deterioration at any point the treatment rationale should switch to damage control. It would therefore seem sensible to adopt a DCR approach in borderline patients until they have proven themselves stable and physiologically resilient [19, 21–23]. This is detailed in Fig. 1.

#### Damage control resuscitation [1, 8, 9, 15, 24, 25]

**Fig. 3:**
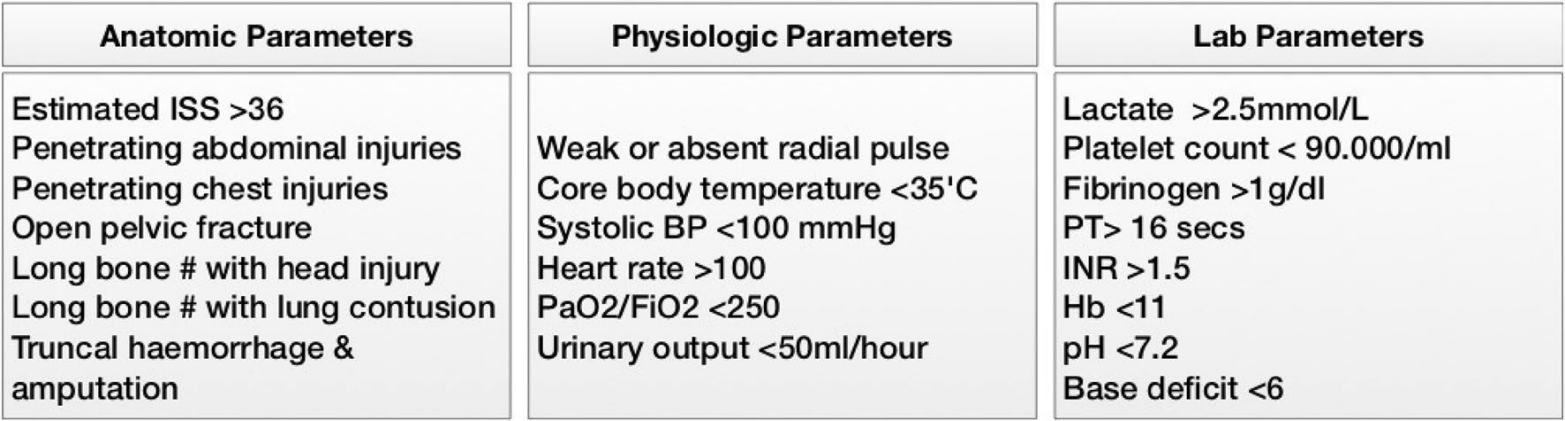
Indications for damage control resuscitation

Specific indications have more recently been described to indicate a damage control approach to resuscitation from the outset. These are summarised in Fig. 3 and describe a series of injury patterns, physiological parameters and lab results that suggest a patient might be at risk. They are particularly worrisome if seen in combination. Care must be taken in those with head injury when considering permissive hypotension. This will detrimentally affect cerebral perfusion pressure and should be avoided, it is discussed below.

### Key components of damage control resuscitation

DCR in trauma differs from traditional approaches by attempting an earlier and more aggressive correction of coagulopathy and metabolic derangements and prioritising haemorrhage control.

The main components of DCR include [1, 26]:

- Permissive hypotension and restrictive fluid administration
- Haemostatic resuscitation
- Rewarming
- Correction of acidosis
- Arrest of haemorrhage by surgical and non-surgical techniques.

Each of these is examined below. It is reasonable to apply individual aspects of this approach initially and escalate or step back as more information regarding injuries and response becomes apparent. For example, in a multiply injured patient with suspected abdominal and pelvic injury, but without initial indicators of massive blood loss, it is reasonable to rewarm the patient as required whilst adopting restrictive fluid resuscitation until more information is available. It should be ensured that blood products are available should they be required. Any deterioration should prompt the use of a Haemostatic resuscitation regime potentially with permissive hypotension whilst arrangements are made for surgical or endovascular haemorrhage arrest.

#### Permissive hypotension and restrictive fluid administration

Traditional fluid resuscitation in the polytrauma patient involved rapid infusion of large volumes of clear fluids in an attempt to rapidly restore circulating blood volume and blood pressure. It has become apparent that this approach has several potentially detrimental consequences. The premise of permissive hypotension is to keep the blood pressure low enough to avoid exacerbating haemorrhage by hydrostatic clot disruption whilst maintaining adequate end organ perfusion. Permissive hypotension and restrictive fluid administration are therefore reciprocal components of this approach; initial fluid administration is delayed or minimised and less aggressive resuscitative end points are used. In practical terms this means targeting systolic blood pressures of 70–90 or 50 mmHg mean arterial. This approximately equates to aiming for the restoration of a palpable radial pulse [2, 11]. Such an approach decreases both the severity and incidence of dilutional coagulopathy, and as such compliments a strategy of haemostatic resuscitation [11]. Second, this reduces fluctuations in, and elevation of, systolic blood pressure which may disrupt the premature blood clot forming in areas of injury causing further bleeding.

The concept of delayed fluid administration in trauma is not new. In the early 1990s a randomised controlled trial of immediate (pre-hospital) versus delayed fluid administration in patients with penetrating truncal injuries demonstrated decreased morbidity (including systemic inflammatory response syndrome, pulmonary oedema and thrombocytopaenia) and mortality in the delayed resuscitation group as well as a shorter hospital stay [27]. The delayed administration group, who received no IV fluid prior to entering the operating room, had lower recorded blood pressures pre-hospital and in the emergency room. Further analysis of this data showed that such an approach might only be advantageous in those with specific injuries, particularly penetrating cardiac trauma, this subgroup benefited most from delayed fluid resuscitation. This study stimulated changes to resuscitative protocols, with consideration given to the timing of fluid administration and resuscitative end points [2, 11].

Whilst having theoretical and practical merits, clear evidence to support permissive hypotension has not been entirely forthcoming. Animal studies appear to support this approach in models of simple exsanguinating haemorrhage and clinical work in other situations, particularly ruptured aortic aneurysm, has demonstrated advantages. Whilst this situation is likely analogous to simple vascular or visceral injury, the situation in trauma is often more complex, particularly in patients with head injury and multiple blunt trauma. In the head injured, the competing interests of maintaining cerebral perfusion pressure whilst keeping the patient from exsanguination can be extremely challenging, hypotension in such patients is generally poorly tolerated. Current advice is to avoid this approach in head injured patients. Further work is necessary to clearly delineate in what situations permissive hypotension should be rigorously applied but as with much of DCR decision making should be individualised [11, 28, 29]. New technologies are allowing novel end points in resuscitation to be investigated which may prove more appropriate than blood pressure in the severely injured. These include near-infrared spectroscopy, measurement of skeletal muscle acid–base status and more sophisticated measures of global acid–base balance [7].

In conclusion therefore, it would appear that restricting initial IV fluid administration in the severely injured should have advantages and the infusion of large volumes of crystalloid is no longer appropriate. In specific situations, permissive hypotension may also be of benefit, particularly in patients with severe haemorrhage from an arterial source. Great caution should be taken in those with concomitant head injury and further work is required to clearly delineate which patients might benefit the most from this approach [11].

#### Haemostatic resuscitation and massive transfusion protocols

Better understanding of the mechanisms underlying coagulopathy in trauma has lead to a paradigm shift in management. Recent protocols attempt to prevent such states occurring in the first place or correct them very rapidly. In terms of restoring circulating volume alone, the type of fluid administered appears to have little consequence. Some advantages to the use of hypertonic saline exist in patients with traumatic brain injury [11, 30, 31]. In general however, there are good reasons to avoid clear fluids and early administration of blood and blood products is generally recommended to replace all the constituents of whole blood from the outset. Recent evidence suggests that timely transfusion, can reduce blood product use overall [7, 32]. The concept centres around the assumption that coagulopathy is present very early after severe injury and rapidly corrective interventions can improve outcomes [2, 33]. This requires us to rethink our concept of massive transfusion and massive transfusion protocols. Massive transfusion has traditionally been defined as those patients requiring more than 10 units of red cells in 24 h. Management of coagulopathy was almost exclusively reactive and only instituted once the patient had received large volumes of blood. Clearly a DCR approach requires the recognition of patients with the potential to require large volume transfusion, this is discussed above [11, 34].

Local protocols will vary but Haemostatic resuscitation aims to deliver a mixture of red blood cells (RBCs), fresh frozen plasma (FFP) and platelets in approximately a 1:1:1 ratio [1]. This is known as balanced transfusion, administration of packed red blood cells being balanced with coagulation factor delivery. Without this, dilution of coagulation factors will exacerbate consumptive loss, this has the potential to rapidly result in a spiral of coagulopathy and worsening blood loss [11]. Whilst such protocols have been found to reduce morbidity and mortality, [35] the requirement for such large amounts of clotting products and the exact composition of this transfusion regime does remain controversial [36]. An observational study recently demonstrated the early survival benefits of delivering clotting product to red cell ratios above 0.5:1 in patients with severe haemorrhage [37]. Conversely, recent military evidence suggests that ratios of a low as 0.35:1 may be preferable [38]. Clearly further work is needed to clarify the situation and it may be that patients with different injury patterns benefit from different regimes. Most major trauma centres have developed their own protocols based upon availability of blood products and local experience [11].

The documented detrimental effects of red blood cell transfusion should also be considered in this context. Blood transfusion has been shown to induce derangements in the immune-inflammatory system with both systemic pro-inflammatory effects and increased infection reported. Volume of blood transfused has been demonstrated to be an independent risk factor for overall morbidity and mortality in trauma patients. Degradation of stored red blood cells during storage has been implicated with decreased red blood cell aggregation and increased release of inflammatory mediators [9]. Further concern comes from the aggressive use of blood products in cases that initially appear to warrant a DCR approach but are subsequently found to be less severely injured than was thought. These patients may receive blood and blood products that were not required at all. The effect of these small volume transfusions is less clear and needs further investigation if the consequences of unnecessary use of blood products are to be understood [39]. Currently therefore, a careful balance must be struck to avoid over zealous use of blood products. Recent evidence would suggest that massive transfusion is still associated with poor outcome and overall use of red blood cells has decreased over time, whilst that of clotting products has increased. This has been associated with a fall in overall mortality [40]. It is important to consider that other factors might explain the observed improvements in outcome.

Advances in laboratory methods mean that when patients do suffer derangement to their clotting mechanism this can now be assessed more rapidly and accurately. Thromboelastography (TEG) provides users with a holistic overview of coagulation through the analysis of platelet function, clotting strength and fibrinolysis. With results available within 20 min, TEG can measure the function of the entire coagulation cascade including platelets, hence simplifying the diagnosis of coagulopathy and guiding further management [2, 19]. Its speed and ability to simultaneously measure multiple aspects of coagulation offer major advantages over standard laboratory methods. It can be used to guide appropriate use of rFVIIa, identify hypercoagulable states and can identify patients at risk for thrombotic events, even when these patients are receiving deep vein thrombosis prophylaxis and have therapeutic concentrations of anti factor Xa [20, 32]. However, TEG is still not widely available and some reliance on traditional methods of assessing clotting function remains [11].

#### Rewarming

Though hypothermia is associated with poor outcome including mortality in multiple trauma patients, the benefits of rewarming remain unclear. Laboratory work has suggested that permissive hypothermia may be beneficial in certain situations, but this has not been demonstrable in clinical practice and early rewarming is still advocated [41–43]. Where hypothermia has not been prevented, it should therefore be reversed. Rewarming may increase vasodilation of peripheral vascular beds, hence improving tissue perfusion, it is recommended that the torso is rewarmed before the extremities to prevent progressive hypotension as a result of this vasodilation. Rewarming can be carried out in the following ways:

1. Passive external rewarming—achieved by warm blankets or increasing room temperature.
2. Active external rewarming—through the use of forced air-warming devices and other heaters.
3. Active internal core rewarming—warming administered fluids and potentially the use of heated oxygen. Warmed bladder and peritoneal irrigation, arteriovenous rewarming and even haemodialysis have all been successfully used in extreme circumstances. Such extracorporeal rewarming techniques are the most efficient, increasing body temperature at a rate of 4–5 °C per hour (compared to only 2 °C by the other aforementioned techniques) [19].

No specific guidelines exist regarding techniques to employ in specific situations, it is important to take steps to prevent hypothermia worsening and identify such states when they occur. Where patients fail to respond to simple measures consideration should be given to more aggressive techniques. When hypothermia is persistent or relapsing, further investigations should be carried out to look for occult on-going haemorrhage [11, 44].

#### Correction of acidosis

The ultimate treatment to reverse metabolic acidosis in the severely injured is the restoration of organ perfusion through volume replacement, permitting acid-base balance to normalise by homeostatic mechanisms. This may be difficult to achieve until haemorrhage is controlled, indeed such measures provide good endpoints to guide resuscitation and persisting abnormalities of acid-base balance should prompt investigation for on-going occult bleeding and hypoperfusion. Whilst it is possible to correct acid-base disturbances pharmacologically, this has more relevance where the cause includes base losses such as in diabetic ketoacidosis. Administration of sodium bicarbonate has been shown to cause the production of carbon dioxide, paradoxically lowering intra cellular pH and leading to a fall in the ionised calcium concentration which has implications for coagulation and cardiac function. Tris(hydroxymethyl)aminomethane leads to a similar effect by accepting hydrogen ions and it’s use has been reported in trauma patients [23]. However, it is widely accepted that correction of metabolic acidosis in these patients is best achieved through aggressive blood and blood product administration alongside appropriate use of vasopressors until the other components of the lethal triad can be addressed [11]. No studies have shown any advantage to pharmacologic management of acidosis in the trauma setting and there are currently no specific guidelines to address the specific reversal of acidosis in the trauma patient [11].

#### Early haemorrhage control

Experience dealing with patients suffering from haemorrhagic shock has demonstrated it is often not possible to restore normal physiology until haemorrhage has been arrested. Indeed, until that point many patients continue to deteriorate despite resuscitative efforts [15]. The speed with which haemorrhage control is achieved is therefore critical. The focus of major trauma protocols on rapid delivery of patients to facilities that allow this are not misplaced [45, 46].

Simple interventions that reduce bleeding before definitive care is available should form part of trauma care resuscitative protocols. Examples of this include the use of pelvic binders, application of compressive dressings to actively bleeding wounds and the use of tourniquets in more severe injuries where this is not effective [47]. These measures should be taken as soon as possible, increasingly in the pre-hospital setting. Tourniquets render the limb ischaemic and may lead to nerve injury, these should therefore be employed with caution. It is important that everything possible is done after application to deliver the patient expediently to appropriate definitive care facilitates. In cases of devastating limb injury, the arrest of life threatening haemorrhage must outweigh any concerns about limb salvage [48]. Temporary aortic balloon catheter tamponade is seeing a resurgence in use [53]. This can be used as a temporising measure in patients with catastrophic abdominal, pelvic and lower limb haemorrhage. Whilst there are significant potential complications and consequences, when simple interventions fail or are not possible, this provides a valid alternative [1]. Many such interventions have been adapted from military practice and further developments are still being seen [49].

The use of tranexamic acid (TXA) in the major trauma setting has been found through multiple studies to reduce the mortality associated with blood loss when administered during resuscitation [27]. TXA functions by blocking the lysine-binding sites on plasminogen and hence inhibiting fibrinolysis, resulting in inhibition of clot degradation [50]. The CRASH-2 trial, showed that the use of TXA could reduce mortality rates associated with exsanguinating haemorrhage by 15 % with few complications. To have such an effect it must be given within 3 h of injury as an immediate intravenous dose of one gram followed by a further 1 g infusion over 8 h. It therefore should form part of early resuscitative protocols. The use of other pro-coagulant therapies remains controversial. Early administration of rFVIIa has been associated with decreased red blood cell use in bleeding patients [32, 51]. Other research has shown a decrease in the transfusion requirements associated with its early use as an adjunct to massive transfusion. However, clinical application remains debatable, with questions remaining regarding the appropriate timing of delivery, selection of patients and the simultaneous use of additional blood components to enhance its effect [32]. The drug appears to be less effective in acidotic patients but remains effective in all but the most severely hypothermic. Recent evidence has suggested an increased risk of subsequent thromboembolic complication following its use [9].

Surgical haemorrhage control is still regarded as the gold standard for the majority of patients and should be rapidly available where required. Open surgery comes with a cost in terms of physiologic derangement however and modern alternatives are increasingly being employed. Endovascular management by interventional radiology allows selective embolisation of bleeding vessels and organs and stent grafting of major and peripheral vessel injuries without many of the specific risks of open procedures [52]. It is often definitive in nature. Temporary intravascular shunts can be life and limb saving by bridging damaged vessels and maintaining blood flow, hence reducing acute haemorrhage and critical warm ischaemia times of distal organs and limbs [1]. As timing of interventions is so critical, interventional radiology facilitates need to be rapidly available 24 h a day to be effective and, this requires organisational development and investment in major trauma centres [54]. It is important to balance decision making and remember that not all bleeding can be controlled non-operatively in a timely manner [55]. Multidisciplinary input is critical and the application of some of these techniques remains controversial [24, 56].

## Conclusions

Understanding of trauma resuscitation has greatly improved in recent years and continues to develop. It is important that the concepts and practicalities of damage control resuscitation are understood by all those involved in the early management of trauma patients and early recognition of those who might benefit from a damage control approach is critical. Given that interventions are so time dependent, it is likely that by developing trauma systems and protocols, further improvements in outcome can be achieved. Research work in this area will allow these approaches to be targetted more accurately to those patients who can benefit most.
